# Uptake, efflux, and toxicity of inorganic and methyl mercury in the endothelial cells (EA.hy926)

**DOI:** 10.1038/s41598-020-66444-5

**Published:** 2020-06-02

**Authors:** Songnian Liu, Martin Tsz-Ki Tsui, Elizabeth Lee, Josh Fowler, Zhenquan Jia

**Affiliations:** Department of Biology, University of North Carolina at Greensboro, Greensboro, NC 27402 USA

**Keywords:** Apoptosis, Environmental impact

## Abstract

Cardiovascular disease (CVD) is the major cause of morbidity, mortality, and health care costs in the United States, and possibly around the world. Among the various risk factors of CVD, environmental and dietary exposures to mercury (Hg), a highly toxic metal traditionally regarded as a neurotoxin, has been recently suggested as a potential contributor towards human atherosclerotic development. In this study, we investigated the toxicity, type of cell death, dose-dependent uptake, and efflux of inorganic HgII (as HgCl_2_) and methylmercury or MeHg (as CH_3_HgCl) in EA.hy926 endothelial cells, as these two forms of Hg are often reported to be present in human blood among the general populations (~20–30% as HgII and ~70–80% as MeHg). Our results showed that HgII is more toxic than MeHg to the endothelial cells, owing to the higher uptake into the cytoplasm and perhaps importantly lower efflux of HgII by the cells, thus the “net” accumulation by the endothelial cells is higher for HgII than MeHg when exposed to the same Hg levels in the media. Furthermore, both HgII and MeHg were found to induce apoptotic and necrotic cell death. This study has important implications for the contributions of these two common Hg species to the development of atherosclerosis, an important process leading to CVD.

## Introduction

Cardiovascular disease (CVDs) is the leading cause of morbidity, mortality, and health care costs in the United States. In 2010 alone, CVDs accounted for 32% of all deaths, or about 1 in every 3 deaths in the United States^1^. Atherosclerosis is the common cause of CVDs, and it results from the build-up of plaques (e.g., fat, cholesterol, etc.) within the arterial walls, narrowing the arterial lumen, and restricting the amount of blood flow. With limited blood flow through these partially clogged arteries, atherosclerosis leads to significant complications, including heart attack, strokes, and aneurysm^2^. Each year, approximately 1.5 million American adults have a heart attack or stroke^3^. Among the various risk factors of atherosclerosis, environmental and dietary exposures to mercury (Hg), a highly toxic metal traditionally labeled as a neurotoxin^4^, has recently been recognized as a potential contributor towards human atherosclerotic development^5–9^.

In fact, the largest concern of Hg is its ubiquitous distribution in the environment as caused by the presence of its inert gaseous phase (as elemental Hg form, or Hg0) and long-range transport in the atmosphere (up to 1–2 years), through wet and dry deposition, making Hg contamination virtually in all environment^10^. There are mainly two forms of Hg in the environment human and wildlife are exposed to–inorganic Hg (*HgII*) and methyl Hg (*MeHg*). There are a number of pathways for the human to accumulate Hg^11^. First, the majority of the population obtain MeHg by consuming fish from uncontaminated (e.g., ocean) and contaminated (e.g., mining-impacted) aquatic environment. HgII in the water and sediment can be converted to MeHg by a specific group of anaerobic microbes, and MeHg can extensively bioaccumulate and biomagnify in the aquatic food webs, often leading to unsafe levels in the long-lived, top predator fish^12^. Second, some populations, especially non-fish eaters, can obtain MeHg by consuming rice, particularly rice products from contaminated and mining regions of the world such as Guizhou province in China^13^, rice obtains MeHg from the flooded soil where active microbial methylation occurs. Third, increasing number of human populations (~15 millions, with ~3 millions as women and children in 70 countries) is exposed to HgII through participating in artisanal and small-scale gold mining (ASGM) in which metallic, elemental Hg (or “quicksilver”) is used to extract gold particles from river sediments in countries such as Peruvian Amazon and Ghana, and workers simply burn off the Hg-gold amalgam in the open air, causing the worker to inhale massive amount of toxic Hg vapor^14^. Similarly, populations exposing to elevated Hg levels include workers in mining and smelting industries and dental health professionals and patients who use amalgam filling^15^.

Based on different monitoring studies, total Hg levels in human blood would be low (e.g., ~2.0 ng/ml) without fish consumption, but increase along with the frequency of fish meal per week (e.g., ~4.8 ng/ml for <2 fish meal/week; ~8.4 ng/ml for 2–4 fish meal/week; ~44.4 ng/ml for >4 fish meal/week in the general, reference populations^16^). However, total Hg levels in human blood can be much elevated in populations with higher Hg exposure such as workers in ASGM operations (e.g., ~102 ng/ml in Ghana^17^). Besides reporting total Hg levels in human blood, a number of studies reporting both HgII and MeHg levels in human blood from the general populations, and the reported percentage of total Hg was 20–30% as HgII and was 70–80% as MeHg^18–20^. Thus, it would be important to evaluate the toxicity and contributions of both Hg forms towards human atherosclerotic development.

Mercury exposure is an increasing health concern, especially global Hg emissions from various sources (especially coal burning and artisanal and small-scale gold mining) are projected to further increase in the next few decades^21^. Previous toxicological studies have mainly focused on the effects of HgII and MeHg on neurodegenerative diseases such as amyotrophic lateral sclerosis, Alzheimer’s diseases, and Parkinson’s disease^4,22,23^. A number of studies also showed that Hg can play a potential pathogenic role in the development of atherosclerosis and its associated hypertension, myocardial infarction and cardiovascular disease^5–9^, however, its underlying mechanism has not been extensively examined. Related to this, a few studies investigated the dietary supplements treating the risk of Hg to the development of CVD^24^.

Human EA.hy926 endothelial cells have been widely used as models for various vascular research because of their endothelial origin and expression of typical endothelial cell surface biomarkers including vascular cell adhesion molecule-1 (VCAM-1) and intercellular adhesion molecule-1 (ICAM-1) and E-Selectin, and they have been widely used as a model system for adhesion assays with several human monocytes^25–27^. The present study was aimed to examine the cytotoxicity of HgII and MeHg, and the types of Hg-induced cell death in the EA.hy926 endothelial cells. We hypothesized that exposure to HgII and MeHg causes vascular damage, in part, by enhancing cell death. This study has further examined the uptake and efflux of HgII and MeHg in the EA.hy926 cells in order to gain further insights into the biokinetics of different Hg species at the cellular levels and to help explain their relative cytotoxicity to the EA.hy926 cells.

## Materials and Methods

### Chemicals and supplies

Dulbecco’s modified Eagle’s medium (DMEM), Hank’s Balanced Salt Solution (HBSS) penicillin, streptomycin, and fetal bovine serum (FBS) were obtained from Gibco-Invitrogen (Carlsbad, CA, USA). 3-[4,5-dimethylthiazol-2yl]-2,5-diphenyltertrazolium bromide (MTT), 2,3-Dimercapto-1-propanesulfonic acid (DMPS), dimethyl sulfoxide (DMSO), glutathione (GSH), and mercury chloride (HgCl_2_) were obtained from Sigma Chemical (St. Louis, MO, USA). Cell scrapper, potassium permanganate, potassium persulfate, trace metal grade (TMG) nitric acid, TMG sulfuric acid, and FITC Annexin V/Dead Cell Apoptosis Kit were obtained from Thermo Fisher Scientific (Waltham, MA, USA). Tissue culture flasks and 48-well tissue culture plates were purchased from Corning (Corning, NY, USA). Methylmercury chloride stock solution (1,000 ppm) was obtained from Alfa Aesar (Haverhill, MA, USA).

### Cell culture

EA.hy926 cells (ATCC, Manassas, VA, USA) were cultured in DMEM supplemented with 10% FBS, 100 µg/ml streptomycin and 100 U/ml penicillin in 75 cm^2^ tissue culture flasks in a humidified atmosphere at 37 °C with 5% CO_2_. The cells were subcultured once they reached 80–90% confluence.

### MTT assay

The cytotoxicity of HgII and MeHg was determined by modified MTT assay as fully described in our previous studies^28–30^. Cells were seeded into 48-well tissue culture plates. After 48 h, the media were aspirated and washed with 0.4 ml of HBSS in each well. Cells then were fed with different concentrations of HgCl_2_ or MeHgCl in fresh HBSS at 37 °C for 24 h. The media were then discarded, followed by the addition of 0.3 ml of HBSS containing 0.2 mg/ml MTT. After incubation of the cells at 37 °C for another 2 h, the media were completely removed. To each well, a mixture of DMSO, isopropanol, and deionized water (1:4:5) was added. The reduction of MTT to formazan by viable cells was spectrophotometrically quantified at 570 nm. Cell viability in treatment was presented as the percentage of MTT reduction in cells as compared to the negative control. Each treatment was conducted in triplicate, and data were reported as means and standard deviations.

### Flow cytometry analysis

Cell apoptosis and cell death induced by HgII or MeHg were measured by FITC Annexin V/Dead Cell Apoptosis Kit assay, with the procedures fully described elsewhere^29,31^. In brief, EA.hy926 cells were treated with various concentrations of HgII or MeHg (0.1–10 µM) for 24 h. Cells were then harvested and washed in cold PBS followed by the re-suspension in annexin-binding buffer and dilution of cell density to ~1 × 10^6^ cells/ml. Then, 100 µl of this solution, 5 µl each of FITC Annexin V and Propidium Iodide (PI) were transferred to a 1.5 ml Eppendorf tube. The cells were gently vortexed and incubated in the dark at room temperature (25 °C) for 15 minutes. After, 400 µl of 1× binding buffer was added to each tube and analyzed by Guava easyCyte Flow Cytometers (EMD Millipore Corporation, Hayward, CA, USA), and the fluorescence of FITC and PI was measured by logarithmic amplification. The number of events analyzed for each gate/sample was 2,000. Each experiment was conducted in triplicate, and data were reported as means and standard deviations.

### Cellular mercury uptake and efflux measurements

EA.hy926 cells were cultured in triplicate 55 cm^2^ petri dish and incubated with DMEM supplemented with 10% FBS. Cell culture media were replaced after 24 h. When cells reached 80–90% confluence, the media were aspirated, and cells were then washed with 8 ml of PBS three times.

For Hg uptake assay, EA.hy926 cells were incubated with various concentrations of HgII or MeHg (0.1–10 µM) in HBSS in a petri dish. At pre-determined time points, media were discarded. Cells were removed by sterile plastic scraper blades from the surface of the petri dish. Subsequently, cells were rinsed with 1 ml of PBS (pH = 7.4). 50 µl of cell suspension was processed for Bradford protein assay. The rest of the cell pellets were collected by centrifugation (10,000 rpm, 5 minutes at 4 °C). Cells were washed with 1 ml of cold (4 °C) PBS in the presence of 1 mM DMPS twice, followed by 1 ml of cold (4 °C) PBS in the presence of 2 mM GSH twice. All the washing solutions were collected in a fresh 15 ml centrifuge tube. Final cell pellets were collected by centrifugation (10,000 rpm, 5 minutes at 4 °C). HgII or MeHg in washing solutions were then regarded as “membrane-bound Hg”, and those in cell pellets were considered as “intracellular Hg”^32^. The amount of total cellular uptake of Hg equals to the sum of these two pools.

For Hg efflux assay, cells were incubated with HBSS containing 100 nM HgII (as HgCl_2_) or MeHg (as CH_3_HgCl) for 2 h (with 9 petri dishes for each Hg treatment). Cells were washed with PBS three times. 8 ml of HBSS was added to each petri dish. Triplicate petri dishes were collected at 0, 1 and 2 h after the start of incubation. Subsequently, cells were processed and washed by the same protocol as described above for the uptake assay. Washing solutions and final cell pellets were processed for quantification of membrane-bound and intracellular Hg, respectively.

### Mercury analysis

In this study, HgII was provided as HgCl_2_ while MeHg was provided as CH_3_HgCl. Since all experiments were performed individually for either HgII or MeHg, we only performed total-Hg analysis to examine the changes in HgII or MeHg levels in samples. Cell pellets containing HgII or MeHg were fully digested by concentrated TMG nitric acid and reagent-grade hydrogen peroxide (4:1; v-v) in a Teflon digestion vessel (Savillex, Eden Prairie, MN, USA) at 80 °C overnight^33^. Digestion of media samples was strengthened by adding an acidic mixture of permanganate and persulfate, and heated at 80 °C overnight^34^. All digested samples were cooled, and neutralized with aliquots of 30% hydroxylamine hydrochloride (Alfa Aesar, Haverhill, MA, USA). Sample Hg was quantified by double amalgamation technique and cold vapor atomic fluorescence spectrophotometer (Brooks Rand Model III, Bothell, WA, USA) as described elsewhere^35^. Throughout the digestion of samples, we included reagent blanks, washing solutions, buffer solutions and a standard reference material (SRM; i.e., National Research Council Canada DORM-4 fish protein). The reagent blanks, HBSS, PBS, PBS + 1 mM DMPS and PBS + 2 mM GSH showed an average of 2.9, 29.7, 30.0, 30.5 and 30.4 pg/ml of Hg, respectively. Digestion of DORM-4 yielded an average recovery of 96.1% of certified total-Hg value.

### Statistical analyses

SAS University Edition was utilized for all data analyses. Analysis of variance (ANOVA) was conducted and differences among means were statistically significant based on calculated p-value (*p* < 0.05).

## Results and Discussion

### Mercury-induced endothelial cell cytotoxicity

**Figure 1:**
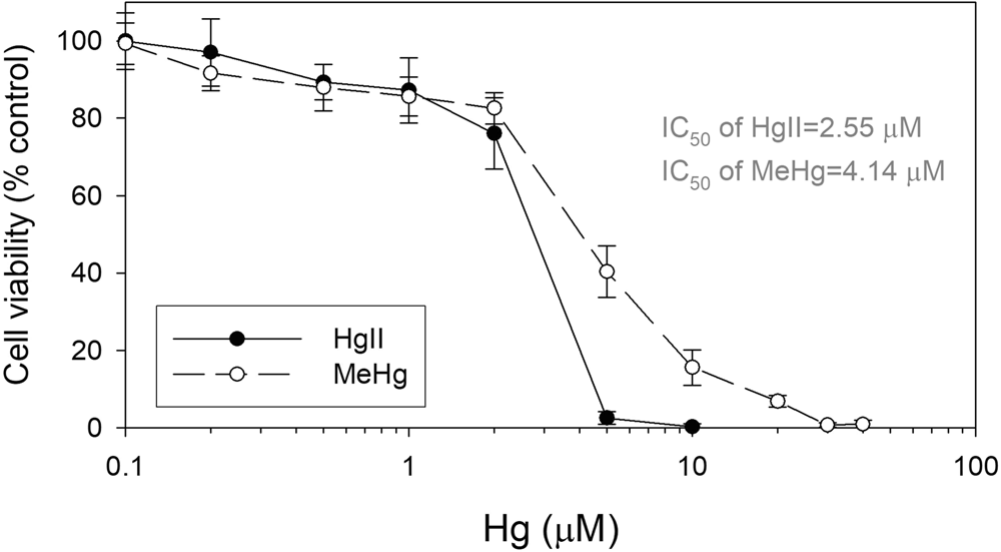
Results of MTT assay of HgII and MeHg on EA.hy926 endothelial cells: Cell viability under exposure to different concentrations of HgII and MeHg for 24 h: dose-response relationship with calculation of median inhibition concentration (IC_50_). Data are mean ± SD (*n* = 3). *p < 0.05 compared to control group (n = 3).

We first investigated the toxic effects of HgII and MeHg on endothelial cell viability. EA.hy926 cells (derived from a permanent human cell line) were treated with a range of concentrations of HgII or MeHg for 24 h, and cellular viability was measured using MTT assay (i.e., testing cell metabolic activity). EA.hy926 cells are known to express the highly-differentiated functional characterization of human vascular endothelium^25^ and are thus widely applied in the study of leukocyte adhesion to endothelial cells. It is interesting to note that different patterns of cytotoxicity are revealed by exposures to HgII or MeHg. As for HgII, no significant cytotoxicity was observed at concentrations ≤1 μM while MeHg became cytotoxic at even lower concentrations (0.2 μM) (Fig. 1), which are likely caused by higher variability (i.e., larger error bar) associated with low levels of HgII exposure (even after repeated experiments). However, at higher concentrations of both HgII and MeHg (5 and 10 μM), we found cell viability was actually much more reduced by exposure to HgII than MeHg.

Thus, the cytotoxic response is “steeper” for HgII exposure than MeHg exposure (Fig. 1). We used probit-log transformation of the data to calculate median inhibition concentration (IC_50_) of cell viability and its associated 95% confidence interval. We found IC_50_ to be lower for HgII (2.55 μM; 95% confidence interval: 1.54–3.56 μM) than MeHg (4.14 μM; 95% confidence interval: 3.11–5.17 μM) (Fig. 1) but the difference is not statistically significant (*p* > 0.05), implying that HgII can be slightly more cytotoxic than MeHg causing cell death as assessed by the MTT assay. It is intriguing that at even much higher concentrations (10 and 20 μM) MeHg exposures did not cause 100% cell death while that would be achieved at only 10 μM of HgII, again revealing some differences between these two Hg species in cytotoxicity.

These concentrations of HgII and MeHg resulting in cytotoxicity are somewhat elevated but also relevant to realistic exposures in human tissue levels. For example, blood concentrations of Hg (mainly MeHg) were reported to be up to ~1 µM (or 200 ng/ml, or ppb) in human subjects with accidental exposure to MeHg and decreased to ~0.13 µM (~26 ng Hg/ml) after 3 months of clearance^36^. Lower, but elevated levels of total Hg in blood were also reported in the general populations with frequent fish meals (~44 ng/ml^16^) and the exposed populations active in ASGM operations (~102 ng/ml^17^). Thus, Hg concentrations ranging from low to ~2 µM for each form of Hg, i.e., HgII and MeHg, were used in this study, which we considered as patho-physiologically relevant and achievable *in vivo*, at least for a short period of time (e.g., 24 h period in our study)^36,37^.

### Mercury-induced apoptotic and necrotic cell death

Apoptosis is a very important phenomenon in order to maintain a constant size and the rates of cell production and cell death^38–40^ while necrosis appears to be the result of acute cellular dysfunction^40,41^. Necrosis is characterized by the ultimate breakdown of the plasma membrane that leads to the release of cytoplasmic contents into the extracellular fluid^40,41^. Also, it triggers the inflammatory response that can lead to further tissue damage by affecting neighboring cells. To examine the types of cell death induced by HgII and MeHg, the FITC annexin V and propidium iodide assay was used to detect the presence of apoptotic and necrotic cells^40,41^. Cell death is partitioned into two main distinct cell death pathways – apoptosis and necrosis. In healthy cells, phosphatidyl-serine (PS) is usually located at the inner leaflet of the plasma membrane. However, during apoptosis, this protein translocates to the extracellular side, and Annexin V is used to bind to PS with high affinity for the detection of apoptosis. Propidium iodide (PI) is a DNA stain, and it is used to distinguish apoptotic from necrotic cells^40^.

**Figure 2:**
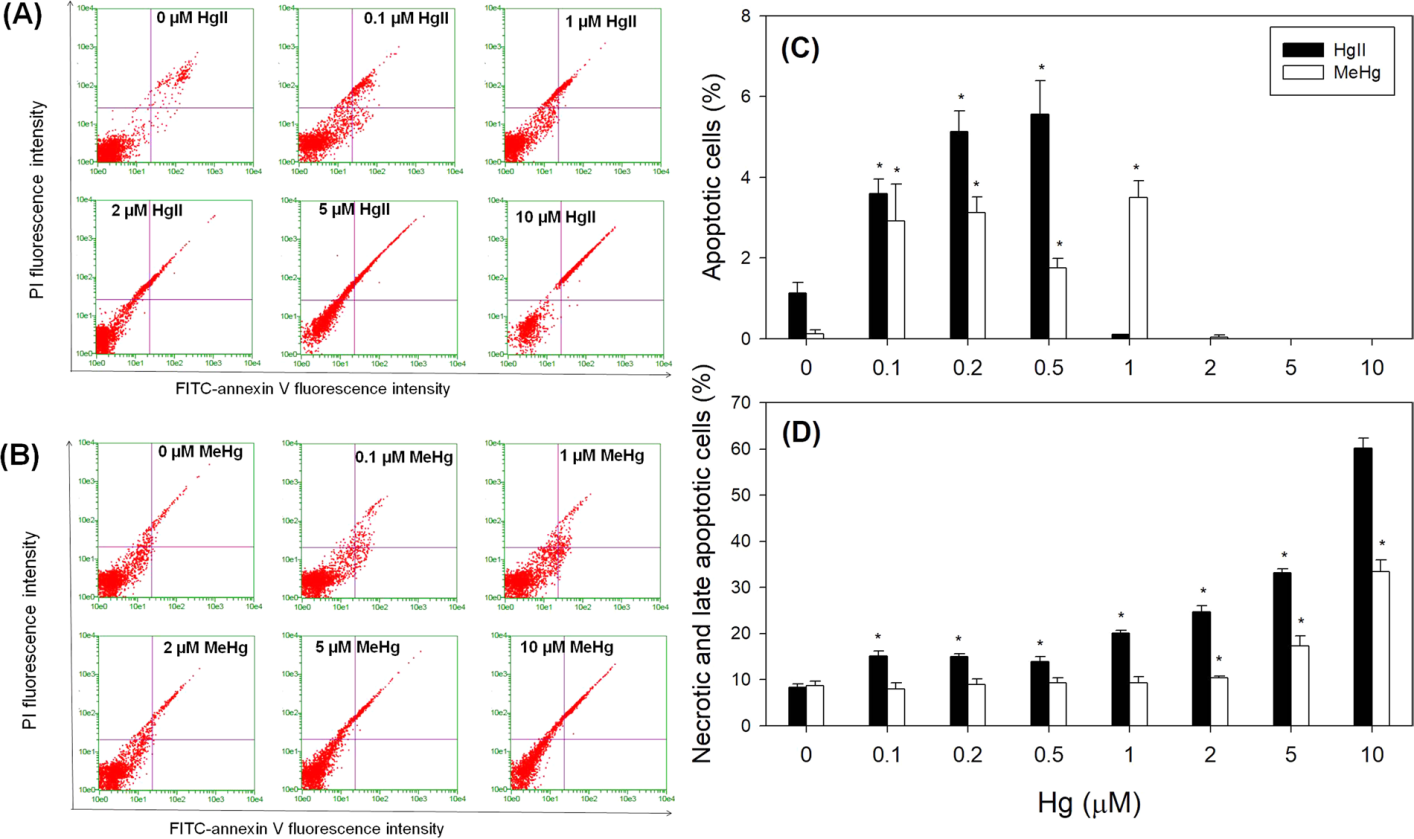
Flow cytometric analysis of apoptotic and necrotic cell population exposed to different concentrations of (**A**) HgII and (**B**) MeHg for 24 h by the Annexin-V staining assay on EA.hy926 endothelial cells. The percent of cells stained as Annexin-V+/PI− (apoptotic cells in the bottom right quadrant) and Annexin-V+/PI+ (necrotic and late apoptotic cells in the upper right quadrant) is presented. Proportion of EA.hy926 endothelial cells upon exposure to different concentrations of HgII and MeHg, and became (**C**) apoptotic cells and (**D**) necrotic cells. Data are mean ± SD (*n* = 3). *p < 0.05 compared to control group (n = 3).

In our study, we found that EA.hy926 cells responded differently to these two Hg species. Specifically, cells incubated with 0.1–0.5 μM of HgII were found to have significantly enhanced apoptotic and necrotic cell populations, especially the latter type (Fig. 2). Beyond exposure to 0.5 μM of HgII, a sharp increase in necrosis and a decrease in apoptosis occurred when HgII level was at 1–10 μM. These results indicated that high levels of HgII caused severe damages on the cell membrane and removed the barrier for the binding of impermeable PI to the nucleus suggesting that necrosis is the predominant form of cell death at elevated concentrations of HgII.

As for MeHg treatment, cells treated with MeHg ranging from 0.2–1 μM were found to lead to a significant increase of the proportion of apoptotic cells (*p* < 0.05; Fig. 2). Interestingly, such concentrations of MeHg did not further increase necrosis compared to the negative control and HgII-treated groups at the same concentration range, indicating that apoptosis is the predominant form of cell death at low levels of MeHg exposure. After 24 h of treatment of 2 μM, 5 μM, and 10 μM of MeHg, EA.hy926 cells had a sharp decline as HgII in the proportion of cell undergoing apoptosis, whereas the percentage of necrotic cells significantly increased. This result suggests that necrosis is the predominant form of cell death at higher concentrations of MeHg, which is similar to that observed for the HgII exposure.

Many studies have demonstrated a causal relationship between a significant proportion of apoptotic and necrotic vascular cell death and the pathogenesis of myocardial infarction and heart failure^42^. The type of endothelial cell death is highly relevant in the stage of development of cardiovascular disease^42,43^. Interestingly, a previous animal study showed that apoptotic and necrotic myocyte cell deaths are independent in contributing to the myocardial damage induced by occlusion of a major epicardial coronary in rats^44^. The frequencies of apoptosis were evaluated by TUNEL that assess DNA fragmentation. The necrosis was detected by a myosin antibody that was administered *in vivo* to determine plasma membrane integrity. The results showed that apoptosis is a relatively rapid process, and it happens within hours from the myocardial damage induced by occlusion of a major epicardial coronary. Following apoptosis, necrotic cell death is the dominant form of myocardial damage leading to the progressive loss of cells with time after infarction. These results revealed that apoptotic and necrotic myocyte cell deaths are independent, contributing to myocardial ischemia reperfusion injury. In our studies, exposure to HgII caused an increase in the necrosis with a concentration-dependent manner with significant effects shown at a concentration of only 0.1 µM (Fig. 2D). However, necrosis was only detected at higher concentrations (>2 µM) when endothelial cells were exposed to MeHg. The expression of Bcl-2 and Fas has been shown to regulate the pathways of apoptosis^45^. Calpain belongs to families of cysteine proteases and plays essential roles in the regulation and execution of necrotic cell death^45^. In addition to Bcl-2, Fas, and calpain, reactive oxygen species (ROS) such as hydrogen peroxide and superoxide have been shown to induce both apoptotic and necrotic forms of cell death^46^. A study by Ghizoni *et al*.^46^ aimed to determine the effects of MeHg exposure on superoxide generation and cellular glutathione (GSH) and toxicity in cultured bovine aortic endothelial cells (BAECs). Results showed that exposure of 1 µM MeHg for 6 h significantly increased superoxide production and decreased levels of intracellular GSH^46^. The BAECs exposed to exposure to 1 μM MeHg also showed a decrease in mitochondrial potential (ΔΨm). Interestingly, the reduction in ΔΨm in mitochondria and an increase in superoxide production induced by MeHg was reduced by co-treatment of NADPH oxidase inhibitor apocynin^46^. This result suggests that MeHg-mediated superoxide production is dependent on the activity of NADPH oxidase resulting in triggering mitochondrial membrane potential disruption and endothelial toxicity. Rat studies have indicated that MeHg exposure can increase superoxide anion production^7^. Mitochondrial studies on rats treated with HgII also showed the increased formation of H_2_O_2_, depletion of GSH, and increase in lipid peroxidation^5^. Further investigation is needed to examine if Bcl-2, Fas and *Calpain* pathways, and ROS and mitochondria direct endothelial cells to induce apoptosis or necrosis.

### Cellular uptake and release of mercury

Chemical is toxic only if it can be taken up by the cells, and thus by merely exposing Hg to endothelial cells may not fully reveal the toxicity mechanism. Here, we sought to better understand the kinetics of uptake and distribution of HgII and MeHg in these endothelial cells (i.e., intracellular *vs*. extracellular distribution). The cell membrane is known to protect intracellular components against the surrounding environment^47,48^. Thus, to determine if HgII and MeHg could be incorporated and maintained or eliminated, cellular uptake and release experiments were performed at the sublethal, realistic level of 0.1 µM (or 20 ng/ml) for both Hg species during a short-term (24 h) exposure.

**Figure 3:**
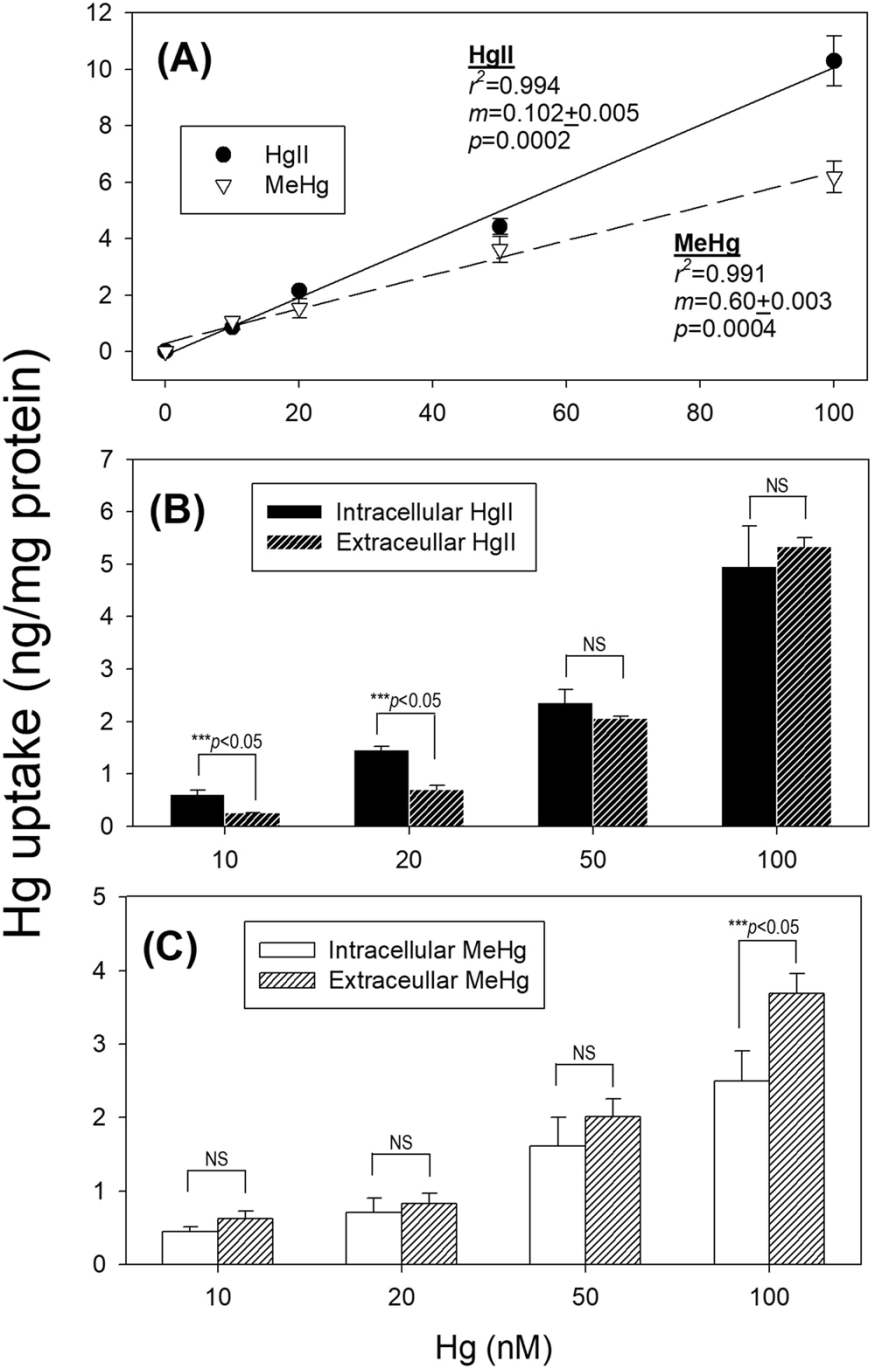
Uptake experiments on EA.hy926 endothelial cells upon exposure to different sublethal concentrations of HgII and MeHg (**A**) relationship between total HgII or MeHg uptake in cells and exposure concentrations after 24 h, (**B**) intracellular and extracellular HgII in cells, and statistical comparison between these two pools of HgII, (NS = not significant, *p* > 0.05), and (**C**) intracellular and extracellular MeHg in cells, and statistical comparison between these two pools of HgII, (NS = not significant, *p* > 0.05). Data are mean ± SD (*n* = 3).

For both HgII and MeHg, we found linear dose-dependent relationships, but we found a significantly (*p* < 0.05) higher slope (*m*) for HgII uptake (mean ± S.E.: 0.102 ± 0.005) than MeHg uptake (0.60 ± 0.003) (Fig. 3A), suggesting that the response of the endothelial cells to the increasing ambient HgII concentrations would be larger, that may help explain the higher toxicity observed in MTT assays at the same concentrations of HgII and MeHg from 5 to 20 μM (Fig. 1).

For cellular uptake, Hg distribution was quantified to distinguish Hg inside cytoplasm (*intracellular*) from Hg bound to cell membrane (*extracellular*) for exposure levels from 10 to 100 nM, except the control (0 nM). For HgII, it is interesting to note that we found significantly more HgII (*p* < 0.05) entering the cytoplasm than Hg bound to the cell membrane at lower levels (10 and 20 nM) but such differences became not significant (*p* > 0.05) at higher exposure levels at 50 and 100 nM of HgII (Fig. 3B). The results may suggest that at higher levels of exposure HgII entering cytoplasm may become limited by the cell membrane, but at lower exposure levels, HgII can enter the cells without such limitations. For MeHg, however, we did not observe significant differences of MeHg inside cytoplasm *vs*. bound to membrane at lower levels (10, 20, and 50 nM) (Fig. 3C), implying that MeHg may be relatively inhibited from entering into the cytoplasm that HgII, and even at higher level (100 nM) we found significantly more MeHg bound to membrane. These uptake results may imply that HgII is more reactive and can enter into the cytoplasm quicker than MeHg, causing higher toxicity as reflected in the MTT assay above (Fig. 1). These results clearly indicated that HgII and MeHg are capable of moving inside the cells, instead of just binding to the outside of the cell membrane.

**Figure 4:**
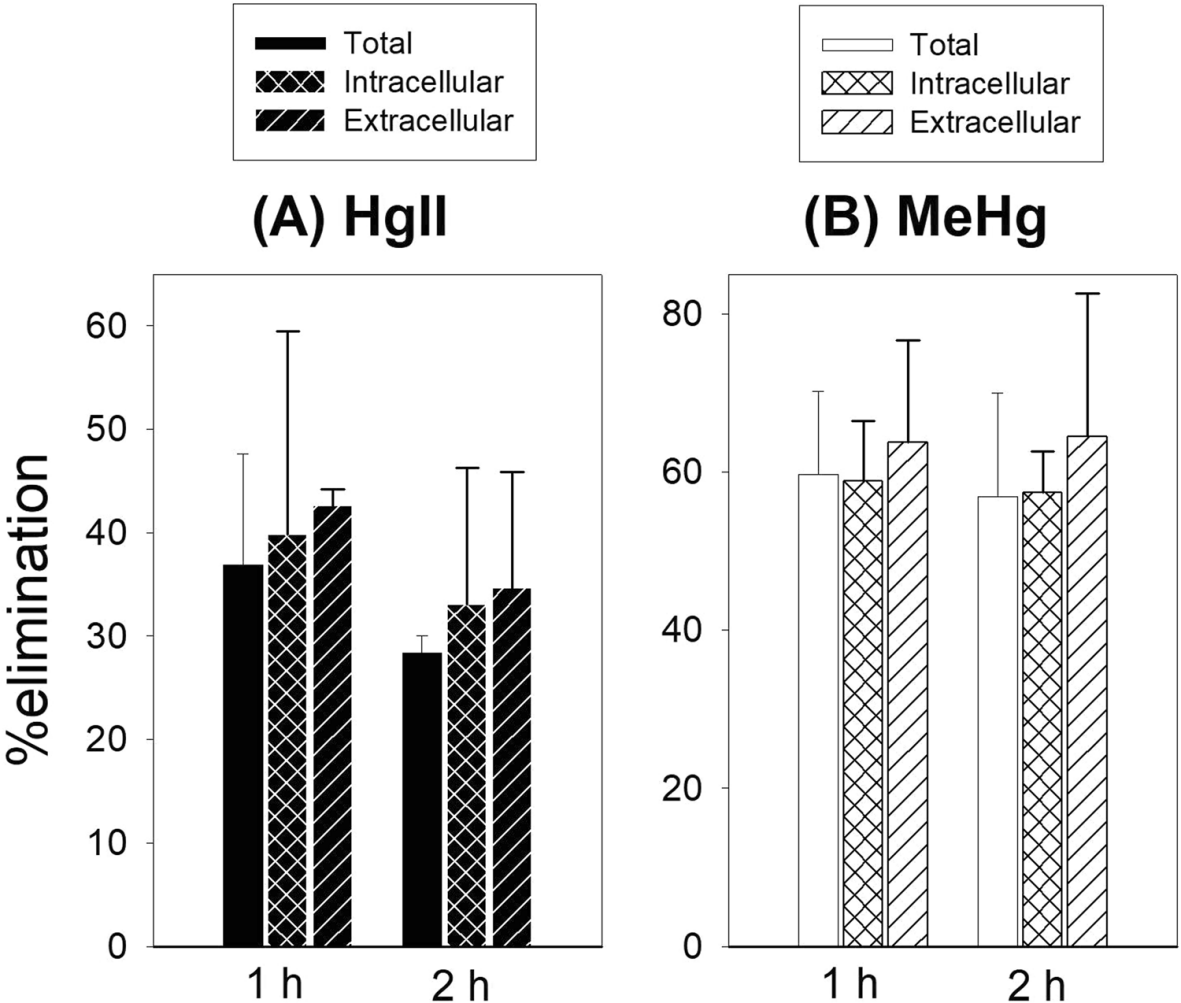
Release experiment of (**A**) HgII and (**B**) MeHg from EA.hy926 cells in time-dependent manner. Data are presented as total, as well as intracellular *vs*. extracellular pools. Data are presented as percent of HgII or MeHg eliminated as compared to HgII or MeHg content in the cells at t = 0 h. Data are mean ± SD (*n* = 3).

After accumulating HgII or MeHg from the exposure media, we transferred and exposed the cells to the HBSS media only to observe their cellular efflux (or release). A significant decrease was found in both HgII and MeHg after only 1 h of release but we observed no significant changes (*p* > 0.05) of HgII and MeHg between 1 and 2 h (Fig. 4). Overall, we found much higher elimination of MeHg than HgII, and for both Hg species we found that higher proportion of membrane-bound Hg (extracellular) was eliminated than intracellular Hg, which support the general notion that chemical binding to the surface of cell would have to overcome lesser barrier (e.g., breaking bonding with ligands) to be released than chemical binding ligands inside the cytoplasm. However, we still observed significant removal of HgII and MeHg from inside the cytoplasm after just 1 h, suggesting that efflux was actively occurring during our 24 h uptake experiment (Fig. 3), and in which we measured the “net” uptake (i.e., uptake minus release). Nevertheless, as we observed positive “net” uptake for both Hg species, implying that the cells are more capable of uptake than efflux of both Hg forms, which suggest that Hg can be efficiently taken up by the endothelial cells upon exposures to both forms of Hg in the bloodstream.

### Implications on Hg cytotoxicity to endothelial cells

To our knowledge, the present study is the first in examining Hg toxicity, pathway of cell death, Hg uptake and release in the endothelial cells. In this context, our results present the direct evidence that HgII and MeHg can enter into the endothelial cells at a concentration as low as 10 nM. Chemical, cellular uptake and release involve crossing the plasma membrane. The cellular membrane is lipid bilayer in nature that provides a physical-chemical barrier in the transport of endogenous and exogenous compounds^47,48^. Previous studies have shown that MeHg can preferentially accumulate in the brain to elicit its neurotoxic effect by targeting on the neuronal cells in the brain^49^. Inside the body, MeHg is readily bound to thiols, and the MeHg-thiol complex can pass the blood-brain barrier via an amino acid carrier to enter neuronal cells^49^. In contrast, HgII is selectively accumulated in the proximal tubules via cysteine uptake transport leading to kidney dysfunction and renal failure^27^. Studies demonstrated that Na^+^-dependent and an Na^+^-independent transporter is critically involved in the uptake of cystine into the proximal tubular epithelia^27^. However, it remains unclear whether the entry mechanism of HgII and MeHg in the endothelial cells is like that in neuronal cells and the proximal tubular epithelial cells.

Previous studies suggested that the presence of cysteine increased the MeHg uptake into the bovine brain capillary endothelial cells *in vitro* (mimic the bovine blood-brain barrier)^50^. Interestingly, HgII can selectively accumulate into the proximal tubules in the segment of the nephron in kidneys via cysteine uptake transport leading to kidney dysfunction and renal failure^27^. However, the roles of cysteine in the Hg uptake into the human vascular endothelial cells remain to be examined in the future.

As a persistent naturally occurring heavy metal and a global pollutant, Hg has become recognized as a priority pollutant in recent decades due to its ubiquitous environmental distribution^10^. In addition to volcanoes, forest fires, crust degassing, many other environmental phenomena and human activities can also contribute to its prevalence in the environment^51^. Indeed, over the last several decade’s emission from anthropogenic sources such as mining, chloroalkali manufacturing, the combustion of fossil fuels, etc. has led to dramatically increased levels of Hg in the environment. Besides its use in industry, Hg has been used in many medicinal compounds, including antibiotics and antiseptics^52^.

While Hg has been studied in the pathology of cardiovascular disease^5–7,53^, however, the biokinetics and toxic action in the cardiovascular system remains largely elusive. Our results showed that the uptake of both HgII and MeHg by EA.hy926 endothelial cells are dose-dependent. Both HgII and MeHg were found to induce apoptotic and necrotic cell death. However, both Hg species behave slightly different with HgII being more “reactive” and toxic to the endothelial cells. This study would increase our understanding on the action of different forms of Hg on endothelial damages and contribute to our ability to assess the cardiovascular risk of human exposure to different Hg forms from different dietary and occupational sources.
